# Maternal adaptation of working mothers with infants or toddlers in South Korea: a systematic review

**DOI:** 10.1186/s12905-021-01357-7

**Published:** 2021-05-21

**Authors:** Jeong-Ah Ahn, Eun Ha Roh, Tiffany Kim, Jin Hyang Lee, Ju-Eun Song

**Affiliations:** 1 College of Nursing, Research Institute of Nursing Science, Ajou University, 164 Worldcup-ro, Yeongtong-gu, Suwon, 16499 Republic of Korea; 2 School of Nursing, Bouvé College of Health Sciences, Northeastern University, 360 Huntington Ave., Boston, MA 02115 USA

**Keywords:** Adaptation, Korea, Mothers, Systematic review, Working women

## Abstract

**Background:**

The fertility rate in South Korea has been decreasing dramatically, as working women postpone or avoid childbirth due to the challenges of maintaining a career while raising a family. Working mothers with infants or toddlers have unique maternal adaptation needs, which must be understood in order to support their needs during childbearing years. Supporting successful maternal adaptation of working mothers is not only essential for each individual new working mother, but also benefits her family, her workplace, and the country.

**Methods:**

A systematic review was conducted to describe the current state of the science on maternal adaptation of working mothers with infants or toddlers in South Korea. Eligible studies, published between 2009 and 2018, were identified by searching electronic databases. Quantitative studies related to the maternal adaptation of Korean working mothers who had a child younger than age 3 years were included. 37 articles met the inclusion criteria for narrative analysis and synthesis.

**Results:**

Studies were classified into 4 major groups by maternal adaptation categories as psychological, behavioral, relational, and cognitive adaptation. The majority of studies were focused on working mothers’ psychological adaptation (n = 36, 97.3%), followed by behavioral (n = 10, 27.0%), relational (n = 9, 24.3%), and cognitive (n = 3, 8.1%) adaptation. We found that maternal adaptation of working mothers was ultimately influenced by diverse variables within their communities, spousal and familial support, personal attributes, and job-related characteristics.

**Conclusions:**

These findings demonstrate the importance of understanding variable aspects of maternal adaptation of working mothers with infants or toddlers. The complexity of working mothers’ needs at the individual, family, and community levels must be considered in order to develop effective intervention programs and public policy for supporting maternal adaptation in Korea.

## Background

The employment rate of Korean married women has increased from 49.8% in 2008 to 57.0% in 2019 [1]. The data show that women’s employment status usually changes in their early twenties and late twenties after marriage and childbirth, reflecting the disruption in women’s careers after childbirth [2]. In recent years, more women of childbearing age have chosen to focus on careers, rather than childbearing. According to Korean national statistics, the total fertility rate (the average number of births that a woman is expected to give in her fertility period) was the highest in the 1960s at 6.0, and then dramatically declined to 2.1 in the 1980s. In 2019, Korea saw the lowest birth rate on record at 0.92 [3]. Although a declining fertility rate is a global phenomenon, this drastic change in Korea’s fertility rate occurred over a relatively short period of time, especially compared to European countries, which took over a century to see such declines.

The rapid drop in birth rates has had an enormous economic and social impact on Korean society, including a declining labor force and rapidly aging population [4]. The country has been struggling to overcome the negative impacts of low birth rates through a multifaceted approach, including interventions to support married and pregnant women during the past decade [5]. However, as employment rates of women continue to increase, the strategies to support women in both career development and childbearing need to be reexamined. Maternal adaptation interventions for working mothers must be both practical and realistic, supporting women’s need to balance both work and family life. Supporting the successful maternal adaptation of working mothers is not only essential for each individual new working mother, but also benefits her family, her workplace, and the country.

The process of maternal adaptation when a woman becomes a mother is described in the literature as a very challenging and complicated process [6, 7]. Previous studies have demonstrated that working mothers suffer significantly more difficulties in maternal adaptation than non-employed mothers [8]. This is “double trouble” for working women, who have parenting stress at home adjusting to family life with a new child, as well as job stress from the workplace [A12]. In particular, in the early stage after childbirth (within 36 months after giving birth), the load of the working mother with infants or toddlers increases, and the burden of maternal adaptation is expected to be greater [7].

Maternal adaptation is defined as a series of processes or outcomes to internalize the diverse tasks of motherhood [9, 10]. According to the literature [10–12], maternal adaptation is a multi-dimensional concept including various types of adaptations as a mother or parent, which are (1) psychological aspect (emotional condition of managing feeling aroused by childcare difficulty and preserving emotional balance), (2) relational aspect (interpersonal adjusting state with partner, child, and parenting supporters), (3) behavioral aspect (performance of childcare behavior), and (4) cognitive aspect (perception regarding parenting or consequences of parenting).

To promote successful maternal adaptation of working mothers with children under 36 months, systematic evidence is necessary to develop a tailored nursing intervention program. Given the complexity of maternal adaptation within Korean society, the ecological system approach is a useful framework for understanding the various interconnected elements of human behavior and development for this concept [13]. The ecological systems theory for human development explains that an individual’s development is influenced by not only an intrapersonal layer but also multiple nested layers of microsystems and exosystems, which are all interconnected with each other [13]. The theory was originally developed to explain child development, but it has also been used in previous studies related to women’s health, and to explain parenting stress and development as a mother [14, 15]. Becoming a mother after a childbirth can be a developmental crisis, and it is influenced by a variety of interrelated factors, which are more easily examined using ecological systems theory [16].

Therefore, the purpose of this systematic literature review is to summarize the current state of the science on maternal adaptation for working mothers with infants or young toddlers in Korea and to analyze various influencing factors of maternal adaptation based on the ecological systems theory.

## Methods

### Study design

The present study was a systematic review to describe the current state of the science on maternal adaptation of working mothers with infants or toddlers in Korea.

### Inclusion criteria

In order to select eligible studies for this review, Population, Intervention, Comparator, Outcomes, and types of Studies (PICOS) were defined. Studies published in the last 10 years (2009–2018) were included if they met the following criteria: (a) population was Korean primiparous or multiparous working women at least 19 years old who gave birth within the last 36 months, (b) studies included any variables in relation to the psychological, relational, behavioral, or cognitive maternal adaptation, and (c) the study design was observational or interventional. Articles were excluded from the review if (a) the studies focused on medical treatment, (b) the primary caregivers were grandparents or fathers, and (c) the studies used qualitative design.

### Search strategy

We searched for articles through the international electronic databases of PubMed and Cumulative Index to Nursing and Allied Health Literature (CINAHL), and Korean electronic databases of Research Information Sharing Service (RISS), Korean-studies Information Service System (KISS), and KoreaMed. We searched the literature using various combinations of Medical Subject Headings (MeSH) terms and/or related keywords covering main search topic/area of maternal adaptation. The keywords included mothers, married women, employment, working mothers, working women, various terms to capture maternal adaptation (maternal identity, maternal role, maternal attachment, maternal behavior, maternal experience, maternal sensitivity, etc.), and Korea.

### Data selection and quality appraisal

2 review authors independently screened titles and abstracts of all studies obtained from the literature search for inclusion in the review, and created a short list of potentially relevant papers. The full text was retrieved if there was any doubt about whether a study should be included in the review. The methodological quality of the articles was first evaluated independently using the Joanna Briggs Institute (JBI)’s Critical Appraisal Checklist by two of the authors [17]. The JBI checklist items on the critical appraisal for observational studies included 5 items regarding research question and design, sampling, instrument, analysis, and presentation of results. Each item was evaluated with either a yes or no. When the total count of yes in the critical appraisal checklist of each study was more than half, the study was included for further evaluation. After discussion, there was complete agreement between the two review authors on the methodological quality of each study. Among 37 studies, all were included in the review after critical appraisal (Table 1).

**Table 1:** Quality assessment of selected studies (N = 37)

Authors [Study ID#]	Item number of critical appraisal					Total score
	1	2	3	4	5	
Kim [A1]	0	1	1	1	1	4
Park et al. [A2]	0	1	1	1	1	4
Lim [A3]	0	1	1	1	1	4
Kim [A4]	0	1	1	1	1	4
Yang and Moon [A5]	0	1	1	1	1	4
Kim [A6]	0	1	1	0	1	3
Kim [A7]	0	1	1	1	1	4
Yang and Choi [A8]	0	1	1	1	1	4
Sung and Park [A9]	0	1	1	1	1	4
Kim and Han [A10]	0	1	1	1	1	4
Park and Moon [A11]	0	1	1	1	1	4
Son [A12]	1	1	1	1	1	5
Lim et al. [A13]	1	1	1	1	1	5
Lee [A14]	0	1	1	1	1	4
Cho and Park [A15]	1	1	1	0	1	4
Joo [A16]	0	1	1	1	1	4
Heo and Kim [A17]	0	1	1	1	1	4
Kim [A18]	0	1	1	1	1	4
Lee and Chin [A19]	1	1	1	1	1	5
Keum and Kim [A20]	1	1	1	0	1	4
Kim and Kwon [A21]	0	1	1	0	1	3
Kim [A22]	1	1	1	1	1	5
Park [A23]	0	1	1	1	1	4
Song et al. [A24]	1	1	1	1	1	5
Lee [A25]	0	1	1	1	1	4
Lee [A26]	0	1	1	0	1	3
Lee [A27]	0	1	1	0	1	3
Choi [A28]	0	1	1	0	1	3
Jeong and Jeon [A29]	0	1	1	1	1	4
Ko [A30]	0	1	1	0	1	3
Kim and Kim [A31]	0	1	1	1	1	4
Kim and Lee [A32]	0	1	1	1	1	4
Yoon and Shin [A33]	1	1	1	1	1	5
Choi [A34]	0	1	1	1	1	4
Kim [A35]	0	1	1	0	1	3
Choi and Jahng [A36]	0	1	1	1	1	4
Kim and Cho [A37]	0	1	1	1	1	4

### Data extraction and analysis

In order to summarize the results of the selected studies, we designed a data extraction form, which included all the major components of each study. For eligible studies, 2 review authors independently extracted the data using the data extraction form. Author(s), study design, theoretical framework, sample, data collection setting, variables, and key findings were extracted and summarized in the form for further analysis and synthesis.

**Fig. 1:**
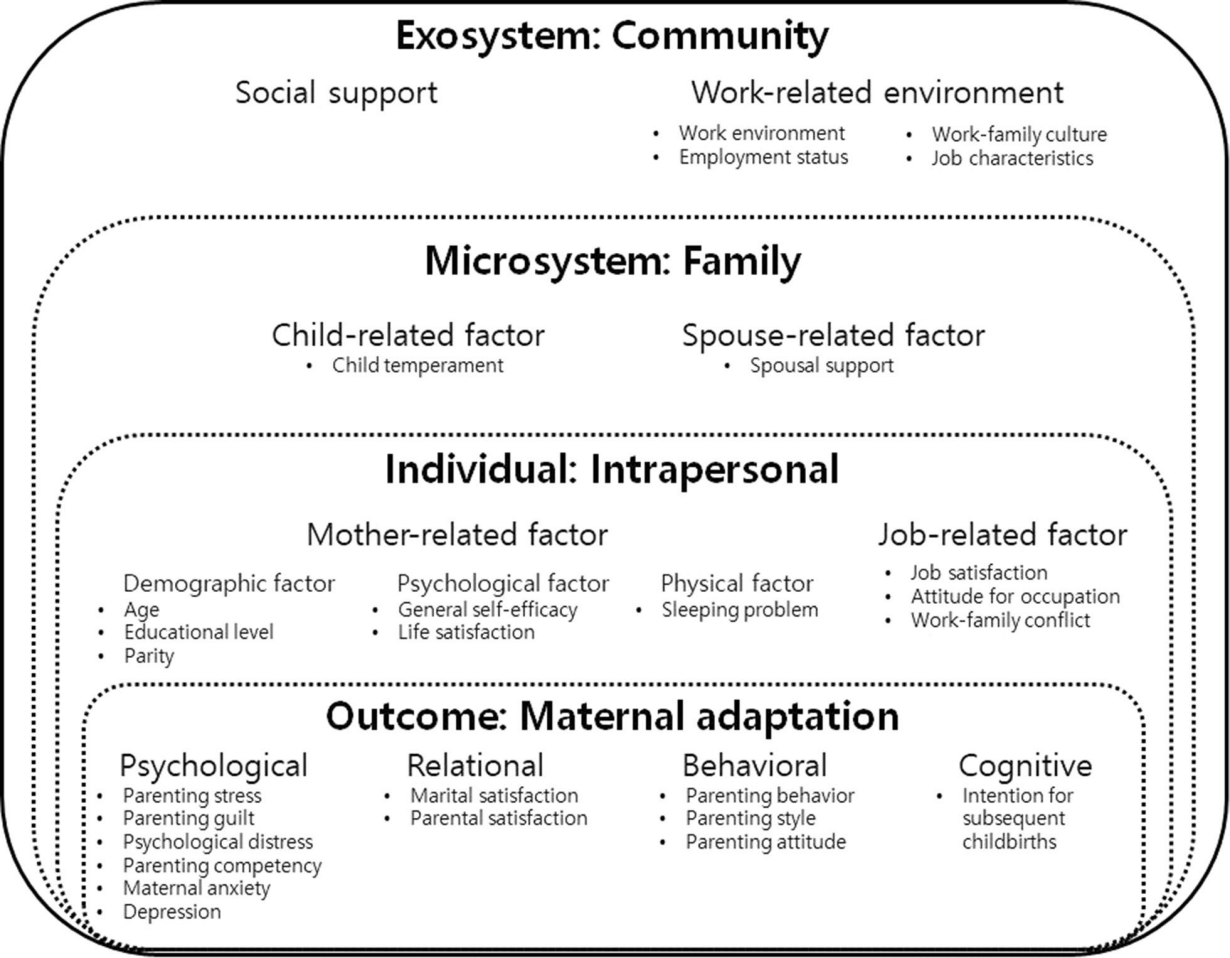
Conceptual model

We performed a narrative synthesis of selected studies’ findings. We classified the major domains of maternal adaptation in the primary studies, and analyzed and deducted conclusions in the systematic review. Then, we derived a path diagram adopting Bronfenbrenner’s ecological systems theory (1979) [13] as the classification criterion for maternal adaptation and diverse related factors from selected studies (Fig. 1). For this path diagram, we selected regression studies (n = 29), which used regression analysis and presented significant impacts (ß-values) on the major concepts regarding maternal adaptation and related issues. We assumed that the dependent variable was maternal adaptation and the explicative factors were diverse related factors. The authors worked together both independently and in collaboration in order to come to a consensus on the classification of the domains and conclusions.

## Results

### Description of studies

**Fig. 2:**
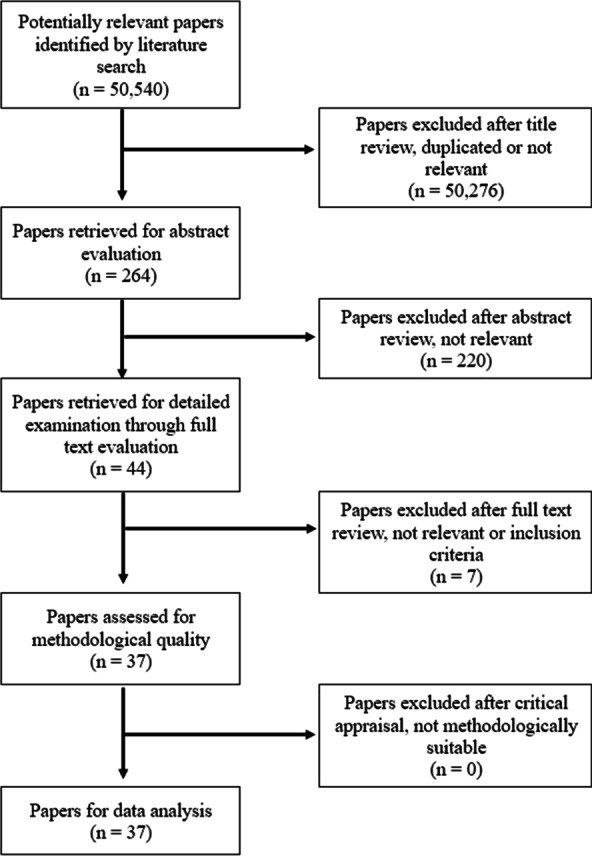
Flow chart

Among 50,540 publications, 37 quantitative studies published between 2009 and 2018 met the inclusion criteria and were selected for the systematic review (Fig. 2).

37 observational studies were included. None of the study authors identified theoretical frameworks to guide their research. The most frequent data collection sites were child-care centers (n = 22, 59.5%). Maternal adaptation was categorized into 4 domains; psychological (n = 36, 97.3%), behavioral (n = 10, 27.0%), relational (n = 9, 24.3%), and cognitive (n = 3, 8.1%) adaptation. Some studies were included in both categories since they dealt with both adaptation domains (Table 2).

**Table 2:** General characteristics of selected studies (N = 37)

Variables	Category	n (%)	Study ID#
Publication year	2009–2013	19 (51.4)	
	2014–2018	18 (48.6)	
Research design	Observational study	37 (100.0)	
Theoretical framework use	No	37 (100.0)	
Setting of data collection	Child-care center	22 (59.5)	
	Panel study on Korean children	6 (16.2)	
Others	9 (24.3)		
Maternal adaptation			
Psychological domain (n = 36, 97.3%)	Parenting stress	27 (73.0)	1–3, 4–6, 11–15, 19–26, 30–37
	Parenting guilt	6 (16.2)	9–11, 16, 18, 29
	Parenting competency	5 (13.5)	7–9, 17, 26
	Psychological distress	3 (8.1)	8, 23, 25
	Depression	3 (8.1)	27, 28, 30
	Maternal anxiety	2 (5.4)	5, 10
Behavioral domain (n = 10, 27.0%)	Parenting style	5 (13.5)	9, 12, 13, 16, 25
	Parenting behavior	4 (10.8)	10, 11, 18, 27
	Parenting attitude	3 (8.1)	12, 13, 28
Relational domain (n = 9, 24.3%)	Marital satisfaction	6 (16.2)	12, 15, 20, 23, 31, 32
	Parental satisfaction	4 (10.8)	7, 9, 32, 35
Cognitive domain (n = 3, 8.1%)	Intention for subsequent childbirths	3 (8.1)	6, 34, 36
Intrapersonal factors			
Mother-related factors	General self-efficacy	2 (5.4)	12,15
Psychological	Life satisfaction	1 (2.7)	7
Physical	Sleeping problem	1 (2.7)	23
Job-related factors	Work-family conflict	4 (10.8)	4, 21, 35, 28
	Job satisfaction	3 (8.1)	20, 34, 36
	Attitude of occupation	1 (2.7)	5
Family-related factors			
Child-related factors	Child temperament	3 (8.1)	12, 14, 26
Spouse-related factors	Spousal support	9 (24.3)	4, 6, 12, 14, 15, 18, 20, 22, 31
Community related factors			
Social support	Social support	11 (29.7)	6, 9, 12, 14, 15, 19, 22, 26, 30, 33, 35
Work-related environment	Employment status	7 (18.9)	9, 10, 22, 23, 27, 32, 33
	Job characteristics	1 (2.7)	23
	Work-family culture	1 (2.7)	29
	Work environment	1 (2.7)	4

Regarding variables in each domain, parenting stress (n = 27, 73.0%), parenting guilt (n = 6, 16.2%), parenting competency (n = 5, 13.5%), psychological distress (n = 3, 8.1%), depression (n = 3, 8.1%), and maternal anxiety (n = 2, 5.4%) were measured for psychological adaptation. Parenting style (n = 5, 13.5%), parenting behavior (n = 4, 10.8%), and parenting attitude (n = 3, 8.1%) were considered for behavioral adaptation. Marital satisfaction (n = 6, 16.2%) and parental satisfaction (n = 4, 10.8%) were evaluated for relational adaptation, and intention for subsequent childbirths (n = 3, 8.1%) was measured for cognitive adaptation (Table 2).

### Maternal adaptation and related factors

**Fig. 3:**
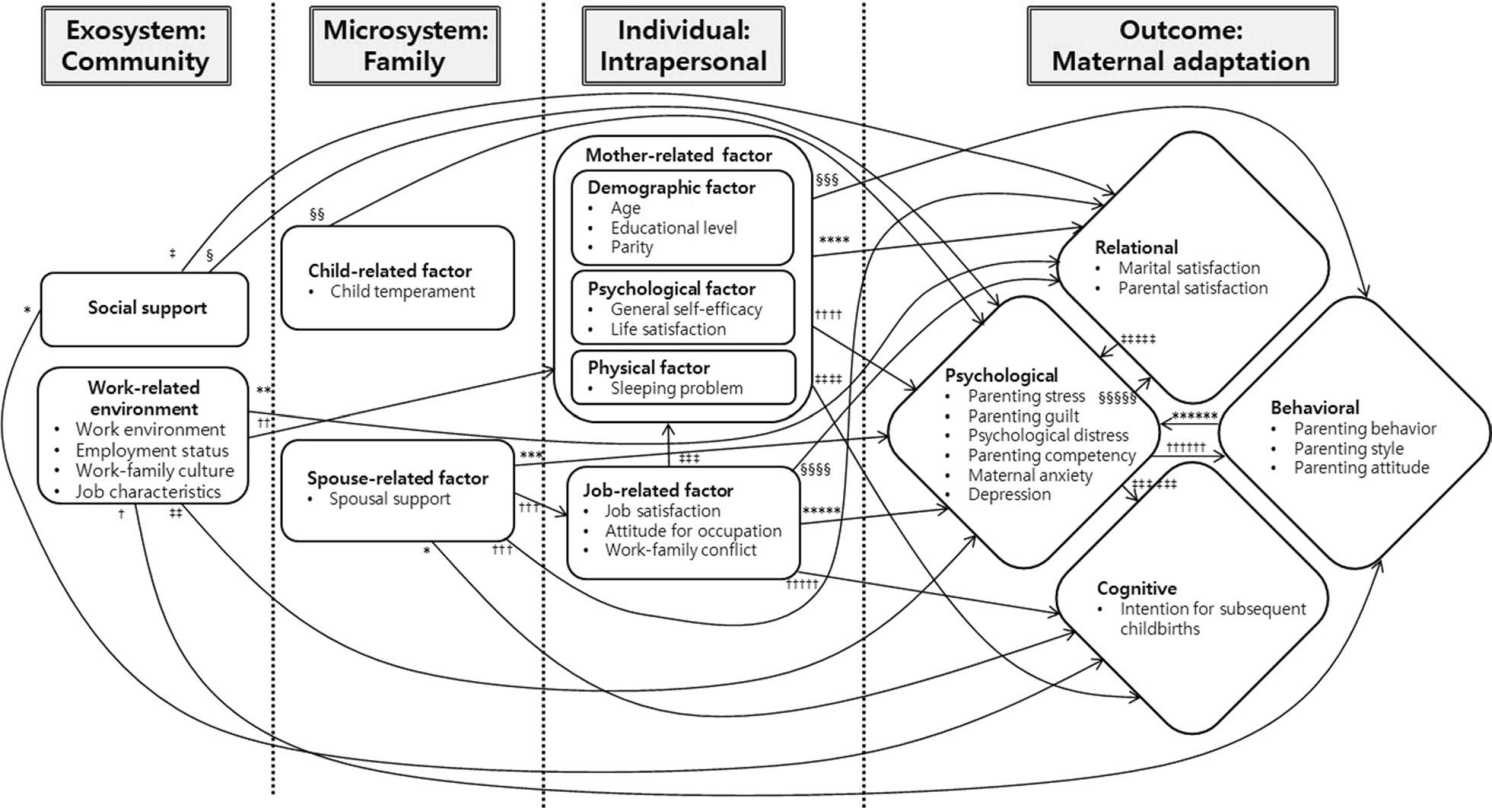
Path diagram. *Kim [A6]. ^†^Sung and Park [A9], Park and Moon [A11], Choi [A28]. ^‡^Cho and Park [A15]. ^$^Cho and Park [A15], Lee and Chin [A19], Kim [A22], Lee [A26], Ko [A30]. **Kim and Kim [A31]. ^††^Kim [A7]. ^‡‡^Kim [A4], Yang and Moon [A5], Kim and Han [A10], Joo [A16], Park [A23], Lee [A27], Choi [A28], Jeong and Jeon [A29]. ^$$^Lim [A3], Son [A12], Lee [A26], Ko [A30]. ***Kim [A4], Cho and Park [A15], Keum and Kim [A20], Kim [A22]. ^†††^Keum and Kim [A20]. ^‡‡‡^Kim [A7]. ^$$$^Park and Moon [A11], Choi [A28]. ****Park [A23]. ^††††^Son [A12], Lee [A14], Park [A23], Song et al. [A24]. ^‡‡‡‡^Kim [A6], Choi [A34], Choi and Jahng [A36]. ^$$$$^Kim and Kwon [A21], Kim [A35]. *****Kim [A4], Yang and Moon [A5], Kim and Kwon [A21]. ^†††††^Choi [A34], Choi and Jahng [A36]. ^‡‡‡‡‡^Lee [A14], Kim and Kwon [A21]. ^$$$$$^Keum and Kim [A20], Kim and Kim [A31], Kim and Lee [A32], Kim [A35]. ******Yang and Moon [A5], Son [A12]. ^††††††^Sung and Park [A9], Lim et al. [A13], Lee [A27]. ^‡‡‡‡‡‡^Kim [A6], Choi [A34], Choi and Jahng [A36]

Maternal adaptation can be categorized by 4 domains (psychological, relational, behavioral, and cognitive adaptation). Figure 3 shows how we propose these domains fit into the ecological systems framework [13]. Each domain is influenced by each nested layer of the intrapersonal (mother-related and job-related) layer, the microsystem (family) layer, and the exosystem (community) layer, and they are also interconnected with each other.

#### Psychological adaptation

Most (36/37) studies investigated the psychological adaptation of working mothers. The measured variables included positive psychological adaptation such as parenting competency, and negative psychological adaptation such as parenting stress, parenting guilt, psychological distress, depression, and maternal anxiety.

Many studies found that working mothers’ psychological adaptation was significantly affected by diverse mother-specific factors such as self-efficacy (ß = − 0.42, *p* < 0.001) [A12], sleeping problems (ß = 0.49–0.56, ps < 0.001) [A23] and job-related factors such as attitude about her occupation (ß = − 0.29 to − 0.27, ps < 0.001) [A5] at the intrapersonal level. Also, psychological adaptation was affected by child-related factors such as child temperament (ß = 0.10, *p* < 0.01 [A12]; ß = − 0.09, *p* < 0.01 [A26]; ß = 0.52, *p* < 0.05 [A30]) and spouse-related factors such as spousal support (ß = − 0.17, *p* < 0.01 [A15]; ß = − 0.32, *p* < 0.05 [A20]; ß = − 0.09, *p* < 0.001 [A22]) at the family level. In addition, psychological adaptation was affected by social support (ß = − 0.12, *p* < 0.001 [A15]; ß = − 0.03, *p* < 0.01 [A19]; ß = − 0.06, *p* < 0.001 [A22]; ß = 0.11, *p* < 0.05 [A26]; ß = − 0.76 to − 0.59, *p* < 0.01 [A30]) and work-related environments such as work-family culture (ß = 0.22, *p* < 0.01 [A4]; ß = − 0.22, *p* < 0.001 [A28]; ß = − 0.21, *p* < 0.05 [A29]) and job characteristics (ß = 0.19, *p* < 0.01 [A5]) at the community level.

#### Behavioral adaptation

10 studies dealt with behavioral adaptation of working mothers. The variables included parenting behavior, parenting style, and parenting attitude. Working mothers’ behavioral adaptation was significantly affected by psychological adaptation, such as parenting stress (ß = − 0.42, *p* < 0.05 [A13]). Also, behavioral adaptation was affected by work-related environments such as work-family culture (ß = − 0.22, *p* < 0.001 [A28]) at the community level. Diverse work-related environmental factors such as flexible working hours benefits in the workplace, paid sick vacation days, fewer working hours, and work-family spillover affected parenting behavior and parenting attitude.

#### Relational adaptation

9 studies identified relational adaptation of working mothers. The measured variables included marital satisfaction and parental satisfaction. Working mothers’ relational adaptation was significantly affected by mother-related factors such as mothers’ sleeping problems (ß = − 0.29, *p* < 0.05) [A23] at the intrapersonal level. Also, relational adaptation was affected by spouse-related factors such as spousal support (ß = 0.35, *p* < 0.001) [A15] at the family level and work-related environment (ß = 0.20, *p* < 0.001) [A7] at the community level.

#### Cognitive adaptation

3 studies presented working mothers’ cognitive adaptation. As cognitive adaptation is the perception regarding parenting or consequences of parenting, successful maternal adaption of working women is associated with positive feelings about subsequent childbirths. For this reason, intention for subsequent childbirths was measured as an indicator of cognitive adaptation in three studies. Working mothers’ job-related factors (ß = 0.19, *p* < 0.01 [A34]; ß = 0.18, *p* < 0.05 [A36]), family-related factors (partner’s help) (ß = 0.16, *p* < 0.05) [A6], and social support (ß = 0.58, *p* < 0.05) [A6] significantly affected working mothers’ intention for subsequent childbirths.

## Discussion

The goal of this systematic review was to summarize and synthesize the research findings on maternal adaptation of Korean working mothers with infants or toddlers. In this review, we selected 37 studies, classified maternal adaptation into 4 domains of psychological, relational, behavioral, and cognitive adaptation, and presented these related factors of maternal adaptation using the ecological systems framework [13].

Working mothers with their infants or toddlers can experience the dual difficulties of fulfilling their roles as mothers (maternal adaptation), but also as an employee in the workplace [A4, A21, A28, A35]. In addition, in this review, we found that their adaptation to these two competing demands are complexly interrelated. The complexity of working mothers’ needs at the individual, family, and community levels must be considered in order to develop effective intervention programs for supporting maternal adaptation in Korea.

Among a total of 37 quantitative studies in this review, there were no experimental studies or studies based on theoretical frameworks. In order to overcome the current low birth rate in Korea, intervention studies focused on supporting maternal adaptation amongst working mothers are needed. These studies must have a robust theoretical foundation that can help make sense of the multiple inter-related and complex factors impacting maternal adaptation in this group [18]. Meleis’ Transitions Theory [19], Mercer’s Maternal Role Attainment Theory [20], and Roy’s Adaptation Theory [21] may be useful in studying maternal adaptation in this population. Further, ecological systems theory is a useful theoretical framework to explain or enhance our understanding of maternal adaptation, because maternal adaptation can be affected by various factors in the nested layers of the ecological model [16]. In the process of maternal adaptation, the early stage after childbirth is a very sensitive and critical period for new mothers [9]. Studies conducted during this critical window will allow researchers to detect conflicts or difficulties in maternal adaptation early and provide more practical interventions for working mothers with young children under 36 months old [22].

Most studies in this review dealt with the domain of psychological adaptation of working mothers, followed by behavioral, relational, and cognitive adaptation. In addition, psychological adaptation was measured by parenting stress, parenting guilt, parenting competency, psychological distress, depression, and maternal anxiety. Parenting stress is widely recognized as the most useful for measuring maternal adaptation of working mothers, as fulfilling parenting roles is accompanied by some level of stress for working mothers [23]. Also, based on our results, psychological adaptation is impacted by diverse mother-related factors (e.g., self-efficacy), job-related factors (e.g., attitude for occupation), child-related factors (e.g., child temperament), spouse-related factors (e.g., spousal support), social support, and work-related environments (e.g., work-family culture). Therefore, some focus areas for nursing interventions should be reduction in parenting stress, parenting guilt, psychological distress, depression, and maternal anxiety of working mothers, considering the various influencing and inter-related factors suggested within this review.

In regard to behavioral adaptation, the current body of literature is focused on parenting behavior, parenting style, and parenting attitude. The behavioral adaptation of working mothers was impacted by various work-related environments (e.g., employment status and work-family culture) and even psychological adaptation such as parenting stress [A34, A36] in this review. The impact of parenting style on a child’s development has also been examined [24]. Future investigators should focus intervention studies on parenting style and parenting behavior amongst working Korean mothers, while considering the affecting factors among the complex variables presented in this review, rather than single factors. Also, this review demonstrates that many aspects of the work environment, such as flexible work hours, paid sick leave, paid vacation days, and so on directly affected the mothers’ behavioral adaptation. Therefore, community-level efforts and strategies for improvement of such work-related environmental factors are necessary.

Relational adaptation included both marital satisfaction and parental satisfaction. Relational adaptation, such as marital satisfaction, is one of the best indicators of maternal adaptation and affected by spousal and social support [A15]. Social or spousal support for mothers is helpful to reduce parenting stress and the negative impact on the maternal role by providing emotional and practical support for mothers [A12]. In particular, spousal support is well known to have a significant impact on mothers’ adaptation and parenting [A4, A15, A20, A22]. Spousal support reduces the burden on the mothers’ child rearing and cause the mother herself to be positively aware of her parenting role [25]. Therefore, promoting spousal support and participation in child rearing should be an important strategy in any maternal adaptation intervention, which should include both parents. In Asian countries, working women still remain the main carers of children and the main contributors to household labor, whereas male partners tend to place greater emphasis on work outside the home. This disparity is particularly stark when children are younger [26]. A cultural shift is needed to impact the household division of labor and the perceived value of parental roles and responsibilities. In addition, it is essential to involve other social support resources, beyond the modern nuclear family. A realistic and systematic social support system should be established by the government to support families where both parents work. Looking internationally, there are many successful models that could be adapted and implemented within Korean society. For example, the European Union and its member countries have implemented various successful policies to support working families and the sharing of parenting responsibilities within society [27]. For instance, the German government offers the Parental Allowance Plus and Partnership Bonus, which provide financial incentives for both parents to work part-time and share caregiving when their children are very young. In addition, high quality and affordable childcare facilities are available to support working families within diverse European countries. The International Labour Organisation has established a long history of legislation primarily focused on the protection of working women during pregnancy and early childhood [28], and inclusion of parental leave has become a symbolic measure of the legislation.

Lastly, as successful maternal adaption of working women is associated with positive perceptions about subsequent childbirths, intention for subsequent childbirths was one of the indicators of cognitive maternal adaptation. Promoting a positive/successful maternal transition experience for working mothers is not only beneficial for the mother/family’s health, but may also increase her willingness to give birth to subsequent children and further contribute to overcoming the historically low birthrate in Korea. Therefore, a tailored intervention program to reduce the ‘double trouble’ working mothers face through improving spousal support at the family level and preparing various strategies for social support or work environmental management at the community level will be essential. At the same time, a qualitative study to explore the changes in cognition related to maternal values and the role of family in Korean society will provide additional direction for future maternal adaptation promotion interventional studies.

This review is limited to working Korean mothers, thus we suggest future studies should be developed to examine the diverse aspects of maternal adaptation and related factors in other populations and cultures, in order further develop the science on maternal adaptation globally. Also, this review only examined maternal adaptation in working mothers, so future studies are needed to compare the differences in the maternal adaptation process between working mothers and non-employed mothers. In this systematic review, we found that the aspect of cognitive adaptation was described in very few articles, and only the intention for subsequent childbirths was presented with significance in the cognitive adaptation in our Figures. Therefore, the cognitive aspect of maternal adaptation needs to be further described with new research. In addition, considering the ongoing COVID-19 pandemic, it will be important to investigate the impacts on maternal adaptation of women who are working remotely from home, young children remain at home at the same time. Despite these limitations, this systematic review is the first to summarize the current literature regarding maternal adaptation based on the ecological systems approach, and it will give rise to a new idea for understanding successful transition to motherhood for working women.

## Conclusions

This study summarized various research studies on the maternal adaptation of working mothers with infants or toddlers in Korea through a systematic literature review. In this review, maternal adaptation was classified into 4 domains: (1) psychological, (2) behavioral, (3) relational, and (4) cognitive adaptation. The relationships among these domains were presented in detail using the ecological framework. This systematic review provides important evidence for researchers, clinicians and policy makers, seeking to design effective maternal adaptation interventions and support working Korean women in their childbearing years.

## Data Availability

The datasets used and analyzed during the current study are available from the corresponding author on reasonable request.

## Appendix: Reference list of selected studies

A1. Kim GN. Relationship between parenting stress and internet shopping addiction in mothers with infants and toddlers. [Master’s thesis] Seoul: Korea University; 2009.

A2. Park YS, Kanzaki M, Park YH, Kim YM. Parenting stress and family functioning of urban mothers with a child less than 3 years of age. Korean J Stress Res. 2009;17(4):349–57.

A3. Lim SH. A study on maternal parenting stress and toddler’s expressive vocabulary. [Master’s thesis] Seoul: Korea National Open University; 2009.

A4. Kim JH. The influence of marital early childhood teacher’s work-family role conflict, husband’s participation in child nurturing, and work environment on parenting stress. [Master’s thesis] Seoul: Kyunghee University; 2010.

A5. Yang SK, Moon HJ. Parental stress of working mother with toddlers: focus on maternal separation anxiety, attitude for occupation, and preschool adjustment. J Korean Home Manage Assoc. 2010;28(4):67–76.

A6. Kim J. The bringing-up stress, social support, re-birth intention of mother with infant or child. [Master’s thesis] Daegoo: Kyungbook University; 2011.

A7. Kim GH. The effects of employed and unemployed mother’s parenting efficacy and parental role satisfaction on life-satisfaction. Fam Environ Res. 2011;49(5):49–57.

A8. Yang EH, Choi HS. A study on the relationships between psychological well-being and parenting self-efficacy of preschooler mothers. J Korea Open Assoc Early Child Educ. 2011;16(6):211–30.

A9. Sung JW, Park SY. Maternal guilt and its related variables: focusing on mother’s employment status and child age. Korean J Child Educ Care. 2011;11(2):123–45.

A10. Kim MH, Han SY. The effects of mother’s separation anxiety and parenting guilt feelings on parenting behavior among working and nonworking mothers. Korean J Hum Dev. 2012;19(4):99–115.

A11. Park HJ, Moon HJ. The effects of mother’s guilty conscience and parenting stress on parenting behavior. J Korean Child Care Educ. 2012;8(2):121–37.

A12. Sohn SM. Parenting stress and related factors of employed and non-employed mothers with infants. J Future Early Child Educ. 2012;19(1):331–57.

A13. Lim HJ, Choi HJ, Lee DK. How emotions during pregnancy and childbirth and parenting stress impact on parenting styles for working moms and homemaker moms. Early Child Educ Res. 2012;32(3):225–44.

A14. Lee YE. Variables on the parenting stress of working mothers with infants. [Master’s thesis] Incheon: Gachon University; 2012.

A15. Cho SH, Park SY. Marital satisfaction of working mothers with infants: The mediating effect of parenting stress. Korean J Fam Ther. 2012;20(3):505–24.

A16. Joo YJ. Exploration of variable related to the parenting guilt of mothers with infants and young children. [Master’s thesis] Incheon: Gachon University; 2012.

A17. Huh HG, Kim MJ. A study of the effects parenting efficacy on the infant playfulness. Korean J Child Educ Care. 2012;12(1):95–110.

A18. Kim EY. Parenting behavior and guilt of the mothers with early child and the rearing support from their husbands. [Master’s thesis] Gwangjoo: Chonnam National University; 2013.

A19. Lee YJ, Chin MJ. Social capital and parental stress of married mothers with young children: Variations by employment status. J Korean Home Econ Assoc. 2013;51(2):229–39.

A20. Keum JH, Kim DS. The causal relationship among the father’s participation in childcare, job satisfaction, parenting stress, and marital satisfaction of working mother. Fam Environ Res. 2014;52(2):141–50.

A21. Kim AN, Kwon YS. Effect of employed mother’s work-mother role conflict on parenting stress: Mediation effect of parental satisfaction. J Digit Converg. 2014;12(10):375–84.

A22. Kim JH. The effect of employment on mothers’ child-rearing stress-focusing on the mediating effects of fathers’ involvement in child-rearing and social support-. [Master’s thesis] Seoul: Catholic University; 2014.

A23. Park HN. Sleep health, psychological distress, parenting stress, marital satisfaction of working mothers according to employment status and working pattern. J Korea Open Assoc Early Child Educ. 2014;19(6):423–41.

A24. Song YJ, Lee MR, Chun HY. Parenting stress changes in both of continuous working and non-working mothers after the birth of their first child: a focus on the effects of the values, knowledge and expectations about their children. Korean J Child Stud. 2014;35(5):15–35.

A25. Lee NS. Mediation effect of the problem-solving styles between psychological distress and parenting stress of mothers with infants and young children: a comparison of working and non-working mothers. J Korean Open Assoc Early Child Educ. 2014;19(2):225–44.

A26. Lee YJ. A study on the influence of the temperament of child, parenting stress and social support on the parenting self-efficacy in employed mothers. Korean J Child Care Educ. 2014;85:117–36.

A27. Lee IK. Impact of depressed mood of working mothers and non-working mothers on the parenting behavior of infant. [Master’s thesis] Incheon: Inha University; 2014.

A28. Choi BR. The impact of working mother’s work-family spillover and depression on parenting. Korean J Soc Welf Educ. 2014;25:99–121.

A29. Jung YJ, Jeon GY. The effects of types of childcare and work-family culture on guilty to the parenting by others of working mothers with infants. Korean J Fam Welf. 2015;20(4):673–91.

A30. Ko EB. Moderation effects of social support on parental stress and depression of working mothers. [Master’s thesis] Seoul: Ewha University; 2016.

A31. Kim D, Kim DH. Moderating effects of marital fondness and admiration and father’s child rearing involvement on the association between parenting stress and marital satisfaction: comparison of unemployed and employed mothers. Fam Environ Res. 2016;54(6):621–9.

A32. Kim JH, Lee JM. The effects of employed and unemployed mother’s parenting stress and parental role satisfaction on marital satisfaction. Couns Psychol Educ Welf. 2016;3(2):29–38.

A33. Yoon SY, Shin N. Stability and reciprocal effects of parenting stress and perceived social support among working and nonworking mothers with young children. Korean J Childcare Educ. 2016;12(6):249–70.

A34. Choi MR. The relationship between parenting stress and intention of second childbirth of working mothers: moderating role of job satisfaction. [Master’s thesis] Seoul: Kyunghee University; 2016.

A35. Kim AN. Mediating effects of social support on relation among parenting stress, work-mother role conflict and parental satisfaction perceived by employed mother with having infant child. J Korea Open Assoc Early Child Educ. 2017;22(5):209–31.

A36. Choi M, Jahng KE. The relationship between parenting stress and second childbirth intention of working mothers with their first child in infancy: The moderating effect of job satisfaction. Korean J Childcare Educ. 2017;13(4):53–73.

A37. Kim YM, Cho YK. Comparison on child raising difficulties and support needs of mothers having infants and toddlers in child care and education centers: based on mothers with and without jobs. J Early Child Educ Welf. 2018;22(3):145–71.

## Abbreviations

PICOS: Population, Intervention, Comparator, Outcomes, and types of Studies
CINAHL: Cumulative Index to Nursing and Allied Health Literature
RISS: Research Information Sharing Service
KISS: Korean-studies Information Service System
MeSH: Medical Subject Headings
JBI: Joanna Briggs Institute
